# Variations in Microstructural and Physicochemical Properties of Soy Wax/Soybean Oil-Derived Oleogels Using Soy Lecithin

**DOI:** 10.3390/polym14193928

**Published:** 2022-09-20

**Authors:** Biswajit Sena, Somali Dhal, Deblu Sahu, Preetam Sarkar, Biswaranjan Mohanty, Maciej Jarzębski, Marek Wieruszewski, Haladhar Behera, Kunal Pal

**Affiliations:** 1 Department of Biotechnology and Medical Engineering, National Institute of Technology, Rourkela 769008, Odisha, India; 2 Department of Food Process Engineering, National Institute of Technology Rourkela, Rourkela 769008, Odisha, India; 3 Department of Pharmaceutics, Institute of Pharmacy and Technology, Salipur, Cuttack 754202, Odisha, India; 4 Department of Physics and Biophysics, Faculty of Food Science and Nutrition, Poznan University of Life Sciences, Wojska Polskiego 38/42, 60-637 Poznan, Poland; 5 Department Mechanical Wood Technology, Faculty of Forestry and Wood Technology, Poznan University of Life Sciences, Wojska Polskiego 28, 60-637 Poznan, Poland

**Keywords:** oleogels, soy lecithin, soy wax, wax crystals, food spectroscopic analysis

## Abstract

Emerging natural-based polymers and materials progress and new technology innovations open the way for unique food products with high nutritional value development. In this regard, oleogel may be essential in replacing fatty acids from food products. In this study, we researched the effects of varied soy lecithin (SYL) concentrations on the various physicochemical characteristics of soy wax (SW)/refined soybean oil (RSO) oleogels. These oleogels had a soft texture. The microscopic analysis of the oleogels suggested that the thickness, length, and density of the wax crystals (needle-shaped) varied as the SYL content was changed. Colorimetric analysis indicated that the oleogels were slightly yellowish. FTIR spectrometry helped analyze the functional groups of the raw materials and the oleogels. All the functional groups present in the raw materials could be accounted for within the oleogels. The only exception is the hydrogen-bonding peak in SW, which was not seen in the FTIR spectrum of the oleogels. It was found that at a critical SYL content, the oleogel showed a stable and repeatable wax network structure. This can be described by the presence of the uniformly distributed fat crystal network in the sample. The DSC analysis revealed that the oleogel samples were thermo-reversible, with their melting and crystallization temperatures ~43 °C and ~22 °C, respectively. In gist, it can be concluded that the incorporation of SYL can impact the color, wax crystal network characteristics, thermal characteristics, and mechanical characteristics of the oleogels in a composition-dependent manner.

## 1. Introduction

Food products can be considered as a complex heterogeneous material, such as a mixture of proteins, polysaccharides, fats, vitamins, antioxidants, microorganisms, dyes, salts, etc. [1,2]. The polymeric (biopolymeric) compounds play an essential role in structuring food products [3]. The interest in food quality and nutrition values has increased strongly in the past couple of years due to an increment in COVID-19 cases. The devastating COVID-19 virus has made us realize the importance of good health. Nowadays, based on “lockdown” experiences and nutritional advice, people are more careful about their food choices and subsequent consumption [4,5]. Readily available foods, including cake, cookies, non-dairy coffee creamer, pizza, burgers, etc., contain a higher amount of *trans* fatty acids (TFAs) and saturated fatty acids (SFAs). This is because such products are comprised of avocado, butter, vanaspati ghee, etc. [6]. Health-conscious persons avoid the consumption of TFAs and saturated fatty acid-containing food products. This is because excessive consumption of TFAs and SFAs leads to hypertension, obesity, and cardiovascular disease (CVD) [7]. As reported by the World Health Organization (WHO), the overall consumption of TFA and SFA should be less than 10% and 1% of total calorie consumption, respectively [8]. In addition, the food should have a higher percentage of mono-unsaturated fatty acids (MUFA) and polyunsaturated fatty acids (PUFA). MUFA and PUFA have been found to have anti-inflammatory and antioxidant effects, which have been linked to a decreased risk of CVD [9]. As lipid-containing foods represent a significant constituent of our diet, modifications of this food can contribute to improving the daily diet [9,10]. Therefore, current researchers are involved with solving the fundamental diet-associated problem. 

Researchers and industries have started focusing on new techniques or products with equivalent functionality as *trans* and saturated fatty acid-rich foods. The equivalent product is expected to maintain the shelf-life, texture, and sensor characteristics of the original food product. In this regard, oleogels with good oil-structuring capabilities could be a better alternative to the usual *trans*- and saturated-fat-structuring approaches. Oleogels are made from vegetable oils, which are free of TFA and have fewer SFAs than “conventional” solid fats (e.g., milk fat or butter, margarine, shortening, tallow, etc.) [11]. Therefore, oleogels have evolved as a substitute for the *trans*- and saturated fats that can help improve the health-conferring activities of food products. Technically, oleogels are defined as semisolids that are composed of oil and oleogelators. Oleogels are comprised of a higher amount of liquid (approximately 90%), with the rest of the solid component known as oleogelator. Oleogelators establish a three-dimensional (3D) structural network, immobilizing the oil and forming oleogels [12]. The oleogelators can form the 3D network by various mechanisms, including fatty acid crystallization, polymeric networks, self-assembled fibrillar networks, and reverse spherical micelles [13]. Some examples of oleogelators include natural waxes (e.g., bees wax, candelilla wax, rice bran wax), stearic acid, ethylcellulose, octadecanol [14], γ-oryzanol+β-sitosterol mixture [15], etc. [16]. Among the aforesaid categories, natural waxes are one of the most effective oleogelators. Natural waxes can create a network of fat crystals with significant oil-binding characteristics, even at low concentrations (lower than 10% *w*/*w*) [17]. The naturally occurring waxes are obtained from seed coats (e.g., rice bran wax (RBX)) [18], plant cuticles (e.g., carnauba wax (CRW)) [19], insect secretions (e.g., beeswax (BW)) [20], or seed-based wax (e.g., soy wax (SW)) [21]. 

In regards to natural waxes, SW is comprised of hydrogenated soybean oil. SW comprises acetylated glycerol esters (e.g., monoacyl and diacyl glycerides) and non-acetylated molecules (e.g., wax ester) [22]. SW is cheaper than other natural waxes, including bees wax [23]. As SW is obtained from soybean oil, it has fewer SFAs, which is good for the health [11]. Among vegetable oils, refined soybean oil (RSO) is only behind palm oil in global production [24]. In addition, it has the best oil yield compared to other vegetable oils. The iodine value of RSO ranges from 120–139 (gI/100 g), which denotes that the oil contains more unsaturation. Unsaturated fatty acids enhance cardiovascular health, reduce cognitive disorders, and prevent osteoporosis [25]. The degumming of crude RSO followed by extraction with an organic solvent (e.g., hexane) yields soy lecithin (SYL) [26]. SYL serves as a crystallization control agent, a wetting agent, a viscosity reducer, an emulsifier, and a releasing agent [27]. At low temperatures, the lecithin molecule arranges the fat crystals differentially depending on the lecithin content [28]. 

New gel systems and compositions are of particular interest in food as well as biomedical applications [29,30]. In this regard, RSO and SW-based oleogels whose properties have been tailored using lecithin can be explored as an alternative for water-based gel systems. The purpose of this study is to modify the characteristics of an optimal RSO-SW oleogel composition by adding SYL. Accordingly, the SYL content in the oleogels was varied to study the changes in the crystal morphology of SW. Further, different properties of the formulated oleogels, including molecular interaction, color, mechanical stability, structural arrangement, and thermal behavior of the oleogels, were studied. For this purpose, we conducted microscopic, colorimetry, mechanical, FTIR, and DSC studies.

## 2. Materials and Methods

### 2.1. Materials

Refined Soybean Oil (Ashoka brand, Shree Hari Agro Industries Ltd., Jaipur, India) was purchased from Amazon, India. Soy wax was procured from Mekasa Products Private Ltd., New Delhi, India. Soy Lecithin was obtained from Urban Platter, Ltd., Maharashtra, India.

### 2.2. Methods

Initially, screening was performed to determine the CGC (critical gelling concentration) of SW for RSO. The CGC was determined by altering the concentration of SW in the range of 1% to 11% *w*/*w*. The oleogel was prepared by melting SW in RSO at 65 °C under mild stirring (300 rpm) for 15 min till a complete homogenous solution was obtained. The homogenous solutions were kept at 4 °C for 90 min for oleogel formation [31]. The said temperature was chosen as fat-based food products are usually stored at this temperature. The formation of oleogel was subsequently validated using the inverted tube technique, which monitored the flow of the samples under gravity [32]. From this screening process, 11% *w*/*w* was found as the CGC of SW as it formed a stable oleogel compared to the lower concentrations of SW. Hence, the oleogel with 11% *w*/*w* of SW was considered the control sample. 

**Figure 1:**
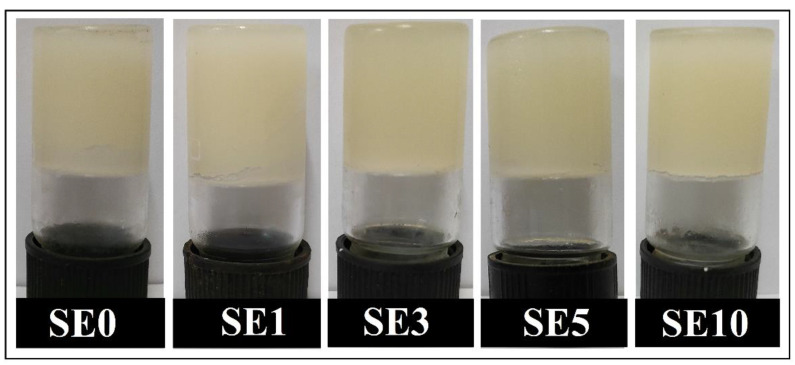
Image of representation of the prepared oleogels.

Subsequently, test samples were prepared. For this purpose, 0.1% (*w*/*w*) stock solution of SYL in RSO was made (SS). Thereafter, RSO, SW, and SS were mixed so that the final concentration of SYL in the 40 g of the test samples was 1 mg (SE1), 3 mg (SE3), 5 mg (SE5), and 10 mg (SE10). The SYL-containing oleogels were prepared in the same manner as the control sample. The composition of the samples is tabulated in Table 1. The prepared oleogels were shown in Figure 1.

### 2.3. Microscopic Study

The samples were imaged using a bright-field microscope (Model: DM750, Leica Microsystems, Wetzlar, Germany). For imaging, a few drops of molten oleogels were put on the glass slide and covered with a cover slip. The molten samples were then allowed to solidify at 4 °C and observed under a microscope. A custom-developed attachment was used to view polarized light micrographs using the same microscope. An ICC 50-HD camera was installed on the microscope for imaging. Both bright-field and polarized micrographs were obtained at 10× magnification.

### 2.4. Colorimetric Analysis

The color of the prepared oleogels was assessed using a custom-built colorimeter. The sensor calibration was initially performed with black and white standard placards. Each sample was placed in a 35 mm Petri plate for this experiment. Color parameters including L*, a*, and b* were measured. The resulting color parameter values were used to determine the whiteness index (WI), yellowness index (YI), and absolute color difference (ΔE). To calculate WI, YI, and ΔE, we used Equations (1)–(3) [33]:

(1) $$\mathrm{WI}=100-\sqrt{{\left(100-L^{*}\right)}^{2}+{\left(a^{*}\right)}^{2}+{\left(b^{*}\right)}^{2}}$$

(2) $$\mathrm{YI}=142.86\frac{b^{*}}{L^{*}}$$

(3) $$\Delta E=\sqrt{{\left(L_{C}^{*}-L_{X}^{*}\right)}^{2}+{\left(a_{C}^{*}-a_{X}^{*}\right)}^{2}+{\left(b_{C}^{*}-b_{X}^{*}\right)}^{2}}$$

where $L_{C}^{*}$,$\;a_{C}^{*}$, and $b_{C}^{*}$ and are the SE0 (control) parameters values. The x tag was substituted for the numerical values obtained for several lecithin-based oleogels. 

### 2.5. FTIR Analysis

An FTIR spectrophotometer (Alpha-E, Bruker, Billerica, MA, USA), operating in the Attenuated Total Reflectance (ATR) mode, was used to record the FTIR spectra of the oleogel samples. The spectrophotometer had a spectral resolution of 4 cm^−1^. At room temperature (25 °C), the solid samples were directly contacted with the ZnSe–ATR crystal and scanned in the wavenumber range of 4000–4500 cm^−1^. The FTIR spectra were obtained for all the samples, including the raw materials. 

### 2.6. Mechanical Study

Static Mechanical Tester (Model: Texture Analyzer HD Plus; Stable Micro Systems, UK) was used to analyze the viscoelastic characteristics of the samples. A 40 g quantity of the samples, kept in a beaker (100 mL), was deformed by 5 mm after a trigger force of 5 g using a 45° Perspex male cone. The speed of the probe was kept at a speed of 1 mm/s. At this set distance (5 mm), the probe was held for 60 s, during which the relaxation profile was recorded. The percentage of relaxed stress (%SR) was calculated using Equation (4) [34].

(4) $$\%\mathrm{SR}=\frac{F_{0}-F_{60}}{F_{0}}\times 100$$

where %SR represents the percentage of relaxed stress, F_0_ represents the maximum peak, and F_60_ denotes the residual force.

### 2.7. DSC 

The thermal properties of the oleogel samples were examined using a differential scanning calorimeter (200 F3 DSC, Maia, Netzsch, Germany). The control and the test samples (~15 mg) were placed in an aluminum pan, sealed with punctured lids. The reference pan was an aluminum pan that was sealed with a punctured lid. The analysis was performed at a scan rate of 5 °C/min in the temperature range of 0–100 °C. The thermograms were analyzed by using Proteus thermal analysis software (NETZSCH, Selb, Germany). During the heating cycle, the samples were heated from 0 °C to 100 °C and subsequently cooled to 0 °C during the cooling cycle. The samples were maintained at 100 °C for 5 min between the heating and cooling cycles.

### 2.8. Statistical Analysis

The experimental measurements were recorded in triplicate and their average values were calculated. The results were expressed as average values along with standard deviations. The final values were represented as mean values ± standard deviation (SD). Statistical data analysis was performed using the student *t*-test in Microsoft Excel. The differences between the samples were considered significant at a *p*-value less than 0.05.

## 3. Results

### 3.1. Microscopic Study

**Figure 2:**
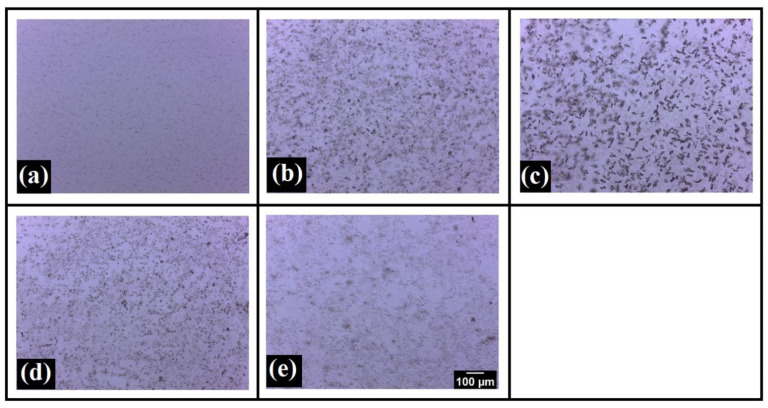
Bright-field micrographs: (**a**) SE0, (**b**) SE1, (**c**) SE3, (**d**) SE5, and (**e**) SE10.

The structural characteristics of the oleogels are usually affected by the size and morphology of the fat crystals. In our study, we added SYL, which acts as a crystal modifier and can alter the microstructure of the oleogels. The wax crystal network produced in the RSO-SW-SYL oleogels by SW was visualized using bright-field and polarized light microscopes. Analyzing such micrographs helps to understand the physical properties of the oleogels. In the bright-field micrographs (Figure 2), it was observed that an increase in the SYL concentration from SE0 to SE3 increased the needle-shaped crystals that appeared as dark patches. In addition, the thickness and length of the needle-shaped crystals increased with the consequent increase in the SYL content. The appearance of the dark patches could be related to the microarchitecture of the wax crystal network [35]. This suggested that adding SYL in lower proportions promoted the formation of needle-shaped wax crystals. With a further increase in the SYL, the size of the needle-shaped wax crystal structures was reduced in SE5 and SE10. This suggests that higher concentrations of SYL hinder the growth of wax crystals. 

**Figure 3:**
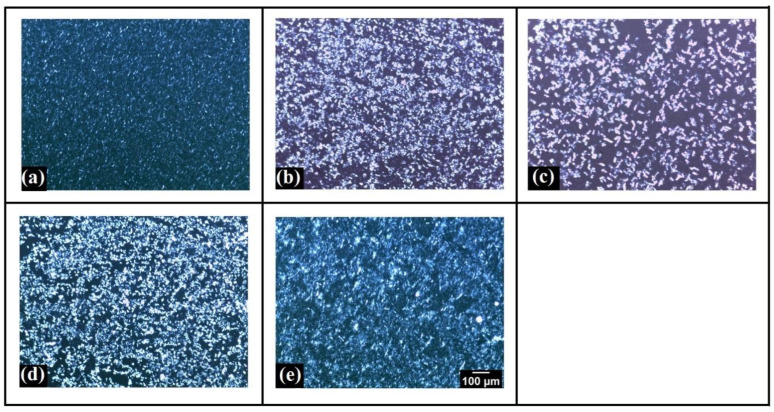
Polarized light micrographs: (**a**) SE0, (**b**) SE1, (**c**) SE3, (**d**) SE5, and (**e**) SE10.

Further, wax microarchitecture was visualized using a polarized light microscope (Figure 3). The crystalline wax molecules had a dazzling white appearance, while the amorphous gel matrix had a dark appearance [36]. Pang et el. (2021) reported this kind of structure, describing the biological fat crystals as birefringent (bright appearance) and the oil phase as optically isotropic (black appearance) [37]. In SE3, there was an increased dark (amorphous) region compared to the rest of the samples. It indicates that in SE3, a highly porous network structure was formed compared to others. In other words, the porosity of the oleogels increased from SE0 to SE3. The size of the fat crystals was also increased as SYL content increased from SE0 to SE3. However, the size of the fat crystals decreased from SE5 to SE10, with a consequent decrease in porosity. This resulted in the formation of a denser structure in SE5 and SE10. Among SE5 and SE10, SE5 showed a more compact microarchitecture of the wax crystal network. These variations are in accordance with previous studies, which suggested that lecithin molecules can act as a crystal habit modifier by changing the shape and size of the wax crystals [38,39]. As per the authors, lecithin may encourage the development of junctions between the crystal molecules, creating a stronger network and entrapping the oil into a firm gel. Hence, it can be expected that the mechanical properties of SE5 would be superior to the other oleogels due to the increase in crystallinity [40]. In gist, the microscopic analysis suggests that the addition of SYL can greatly affect the wax crystal morphology and the microarchitecture of the wax crystal network of the oleogels.

### 3.2. Colorimetric Analysis

**Figure 4:**
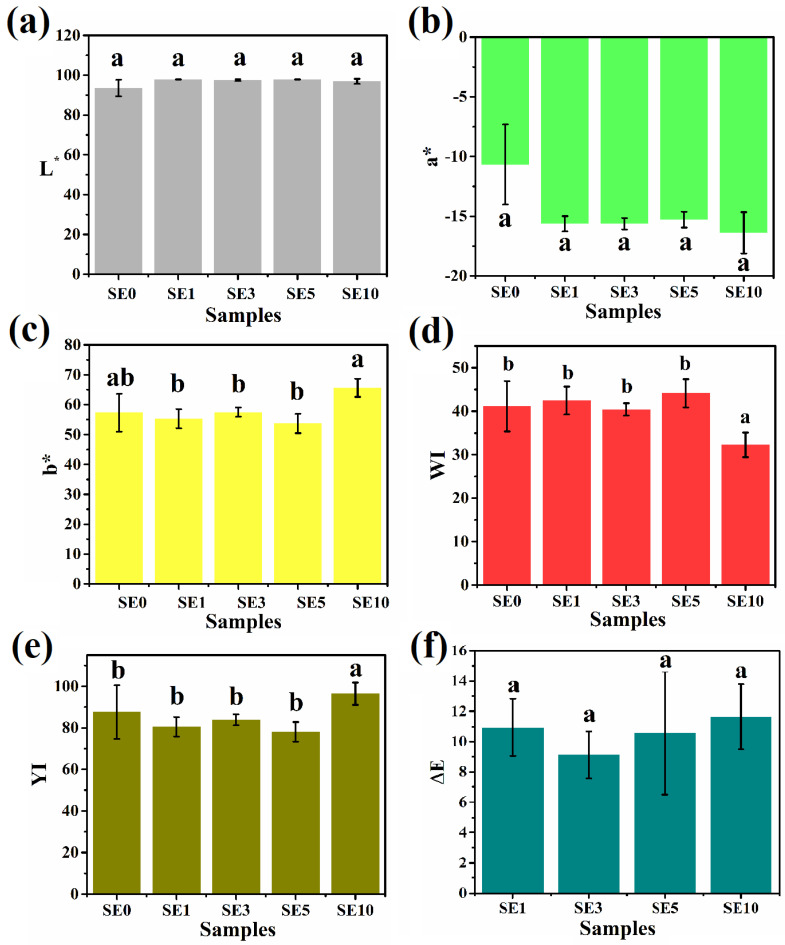
Color parameters: (**a**) L* values, (**b**) a* values, (**c**) b* values, (**d**) Whiteness index values, (**e**) Yellowness index values, and (**f**) ΔE values. The values denoted in the graphs are the average ± SD of triplicate samples (Significance level, *p* = 0.05). Bars with different letters differ significantly (*p* < 0.05).

Color, in addition to flavor and texture, is an essential factor in determining the sensory qualities of food products [41]. As a result, color is an important component of visual appearance, influencing and underpinning assumptions about the predicted smell and taste [42]. The presence of various ingredients used to make the oleogels governs their final coloration. Previous research, for example, found that olive oil oleogels differed significantly from the color of virgin olive oil [43]. Accordingly, the color scales are widely utilized in the food sector. The CIE Lab color scale is considered a standard scale for assessing the psychometric index of lightness (L*), which varies from black (0) to white (100). The chromaticity coordinates, i.e., the a* and b* components, have no specific numerical limits [44]. The a* component ranges from green (negative) to red (positive), whereas the b* component ranges from blue (negative) to yellow (positive) [45]. Figure 4a shows that the average L* values of all the samples was higher than 90. This suggested that the prepared oleogels were luminous. It might be due to the presence of uniformly distributed smaller fat crystals (as seen in Figure 3) that reflect more incident light and become luminous [46]. It is desirable to have higher L* values in food products as it helps to improve the food color perception [47]. The variation of the chromatic components of the formulated oleogels is demonstrated in Figure 4b,c. It can be observed that the samples acquired green and yellow tones, respectively [48]. The yellow tone of the prepared samples might be due to the influence of SYL, which is slightly yellowish [49]. Insignificant statistical differences (*p* > 0.05) existed between the L* and a* values of all the prepared samples. The b* component of the SE0 sample was similar to the rest of SYL-added oleogel samples (*p* > 0.05). However, the b* value of the SE10 was significantly higher than other SYL-containing samples (*p* < 0.05). 

Further, the WI and YI values of the oleogel samples were calculated (Equations (1) and (2)). The WI is a measure of the degree of perfect whiteness and is typically based on the blue–yellow dimension [44]. WI combines L*, a*, and b* values into a single term. The WI of the samples was observed to be in the range of 32 to 45 (Figure 4c). This signifies that the samples were slightly darker in color. This might be due to the presence of a slightly yellow tone [44]. The WI value of SE0 was similar to SE1, SE3, and SE5. Furthermore, the higher SYL concentration in SE10 showed a significant drop in the WI value than in SE0 (*p* < 0.05). Among all SYL-added samples, the variation of WI values in SE1, SE3, and SE5 was insignificant (*p* > 0.05). The WI value of SE10 was lower than the rest of the SYL-added samples. 

Thereafter, the YI of the samples was calculated by combining the L* and b* values (Equation (2)). Similar to the WI values, among all SYL-added samples, the YI values of SE1, SE3, and SE5 were identical (*p* > 0.05). Nevertheless, in SE10, there was a significant increment in YI value from the rest of the SYL-added samples (*p* < 0.05). 

Lastly, the absolute color difference (ΔE) value was calculated using Equation (3). The ΔE value is always calculated concerning a particular standard, and the color difference information is represented as numerical values (Figure 4f). In our case, we have considered SE0 as the standard. The ΔE values were found to be in the range of 4 and 12, suggesting that the color difference among the samples can be identified by the naked eye [50]. However, ΔE values of the SYL-added samples were similarly valued (*p* > 0.05). 

### 3.3. FTIR Analysis

**Figure 5:**
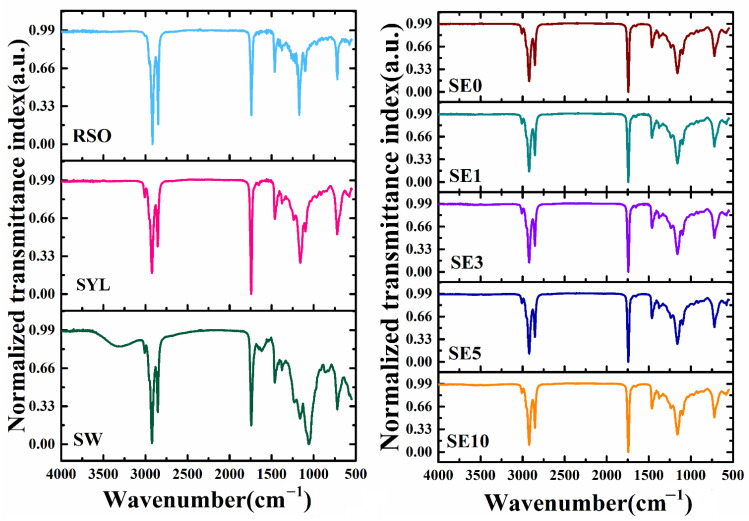
FTIR spectra of the raw materials and oleogels.

The FTIR spectrum revealed probable interactions between functional groups of compounds that are included in the oleogel samples (Figure 5). The spectral information of the prepared oleogels was collected in the wavenumber range of 4000–4500 cm^−1^. The major component of the oleogels was RSO, which showed peaks at 3009, 2916, 2848, 1740, 1463, 1377, 1173, 1097, 720, and 575 cm^−1^. The shoulder peak at 3009 cm^−1^ represents the C–H stretching symmetric vibration of cis-olefinic double bonds (=CH) [51]. This peak can be related to the linoleic/linolenic acyl groups present in the fatty acids of RSO [52]. The peaks at wavenumbers 2916 and 2848 cm^−1^ appeared as a sharp dual peak in the spectrum of RSO. The peak at 2916 cm^−1^ can be attributed to the –CH_2_ stretching vibrations and the peak at 2854 cm^−1^ signifies symmetric stretching vibration of C–H of aliphatic CH_2_ functional groups [53]. The strong and single peak at 1740 cm^−1^ was due to the C=O stretching vibrations. The C=O stretching vibrations can be associated with the ester carbonyl (C=O) functional groups of triglycerides and the carboxylic groups of fatty acids [54]. At 1465 cm^−1^, peaks of –CH_2_ and –CH_3_ were also observed for RSO. This peak is due to the C–H bending vibrations in n-alkanes [55]. The small peak at 1377 cm^−1^ can be ascribed to the C–H symmetric bending vibrations of CH_2_ groups. The peaks obtained at 1173 and 1097 cm^−1^ can be assigned to the C–O stretching of the ester and –CH deformation vibrations of fatty acids, respectively [56]. In the extreme right of the spectra of RSO, a small peak was present at 720 cm^−1^. The occurrence of this peak can be associated with bending vibrations of cis C=C groups of disubstituted olefins and CH_2_ rocking vibrations [52]. 

The SYL-spectrum was similar to that of RSO, except that the peak at 3009 cm^−1^ had a higher intensity in SYL compared to RSO. SYL typically contains about 5% complex sugars, 11% glycolipids, 18% phosphatidylcholine (PC), 14% phosphatidylethanolamine (PE), 9% phosphatidylinositol (PI), 5% phosphatidic acid (PA), 2% other phospholipids, and 37% neutral oil [57,58]. SYL is, therefore, mostly comprised of phospholipids (PE, PA, PC, and PI), which typically have two hydrophobic fatty acyl chains and a polar hydrophilic head group. Such groups are responsible for the peaks obtained in the FTIR spectrum of SYL. Because of the major molecular resemblance between lecithin and triglycerides, the FTIR spectra of SYL show that there is a substantial spectrum overlap between SYL and RSO [59]. 

Further, the SW spectrum was similar to that of RSO and SYL, except for a few extra peaks in SW. The presence of a broad peak at 3312 cm^−1^ can be ascribed to the O–H stretching vibrations. SW and other vegetable waxes are comprised of long-chain fatty acids and saturated hydrocarbons [60]. Natural waxes consist of sterols, sterol esters, and long-chain alcohols, which give rise to hydroxyl group stretching vibrations [61]. 

The spectra of the oleogel samples were similar to that of the RSO. The characteristic peak of the SW at 3312 cm^−1^ was absent in the oleogel samples, suggesting the absence of hydrogen bonding in the oleogel samples. This can be reasoned by the presence of RSO in high amounts compared to SW and SYL. However, there might be some hydrophobic interactions between the non-polar oil molecules and the hydrophobic part of lecithin molecules [62]. Further, the presence of unsaturation in the prepared oleogels was confirmed by the peak at 3009 cm^−1^. The absorption band in the range of 1500 and 900 cm^−1^, known as the “fingerprint region”, was similar in all the oleogel samples. 

### 3.4. Mechanical Studies

**Figure 6:**
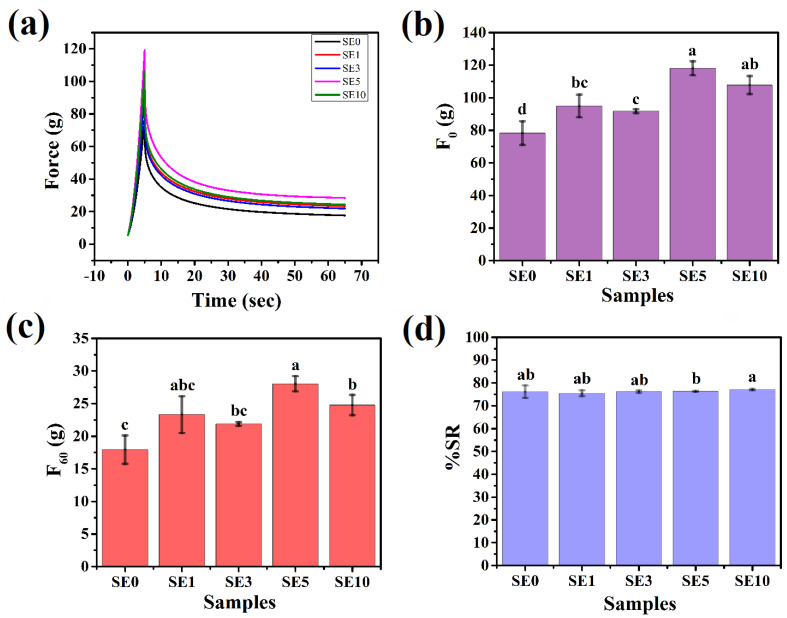
Mechanical parameters: (**a**) Stress relaxation profile, (**b**) F_0_ values, (**c**) F_60_ values, and (**d**) %SR values. The values denoted in the graph are the average ± SD of triplicate samples (Significance level, *p* = 0.05). Bars with different letters differ significantly (*p* < 0.05).

The stress relaxation study was performed to evaluate the viscoelastic characteristics of the oleogels. The relaxation profiles of the oleogels are depicted in Figure 6a. As reported in [63], the total stress sensed by the test probe is the combination of the stress exerted by the wax network and the pressure created by the entrapped oil. The highest force attained in the stress relaxation curve is used to forecast the firmness (F_0_) of the samples. After reaching the maximum force, the stress is maintained for a particular duration, known as holding time. In our case, the holding time was 60 s. During this holding time, the force values decreased exponentially and attained the minima, i.e., residual elastic force (F_60_). Compared to SE0, the inclusion of SYL in the samples resulted in a significant increase in the F_0_ value (*p* < 0.05). In other words, the F_0_ value of SE0 was the lowest. Among the SYL-containing samples, SE1 showed similar F_0_ values to that of SE3 and SE10 (*p* > 0.05). However, a significant increase was observed in SE5 (*p* < 0.05). Further addition of SYL in SE3 resulted in the lowest F_0_ value among the SYL-added sample. The decrement in the F_0_ value of SE3 from SE5 and SE10 was significant (*p* < 0.05). Furthermore, an increase in SYL content in SE5 showed the highest F_0_ value. Interestingly, it was observed that the F_0_ value of SE10 was statistically similar to that of SE5. Therefore, it can be hypothesized that the firmness of the oleogels was more pronounced at a higher SYL content. This might be due to the formation of a dense wax crystal network in SE5 and SE10, as observed from the polarized light microscopy. It was noticed that SE5 showed a minimal standard deviation compared to other SYL-added samples. This suggested that there is a possibility of a stable and repeatable wax network structure in SE5 [33].

The residual elastic force resulting from the gelator crystal rearrangement in the three-dimensional gelator network causes the force to decay to its minimal value [64]. A higher F_60_ value is associated with a rigid crystal structure [65]. The F_60_ value of SE0 was the lowest. SYL inclusion in SE1 and SE3 showed a similar F_60_ value to that of SE0 (*p* > 0.05). Thereafter, a further rise in SYL in SE5 and SE10 showed a significant increment in the F_60_ values compared to SE0. Among all the SYL-added samples, SE1 showed a similar F_60_ value to the rest of the samples. Compared to SE3, SE5 showed a significant increase in the F_60_ value (*p* < 0.05). The F_60_ value of SE5 was the highest (*p* < 0.05). SE10 showed a similar F_60_ value to that of SE3. The variation in the F_60_ value in SE10 compared to SE5 was significant (*p* < 0.05). In gist, SE5 showed greater rigidity than the others, possibly due to the uniformly distributed wax crystal inside the oleogel matrix (Figure 6c). 

The %SR can be defined as the ability to absorb energy during strained conditions. The percentage of stress relaxation is calculated using Equation (4). The %SR of SE0 was similarly valued to the rest of the SYL-added samples (*p* > 0.05). SE1, SE3, and SE10 samples showed similar %SR values. The %SR value of SE5 was similarly valued to SE1 and SE3 (*p* > 0.05); however, the same was significantly lower than SE10 (*p* < 0.05). In summary, the addition of SYL in the RSO-SW oleogels did not greatly affect the viscoelastic characteristics of the oleogels. In addition, since the %SR values of all the oleogels were higher than 70%, the oleogels were predominantly elastic. 

### 3.5. DSC Analysis

**Figure 7:**
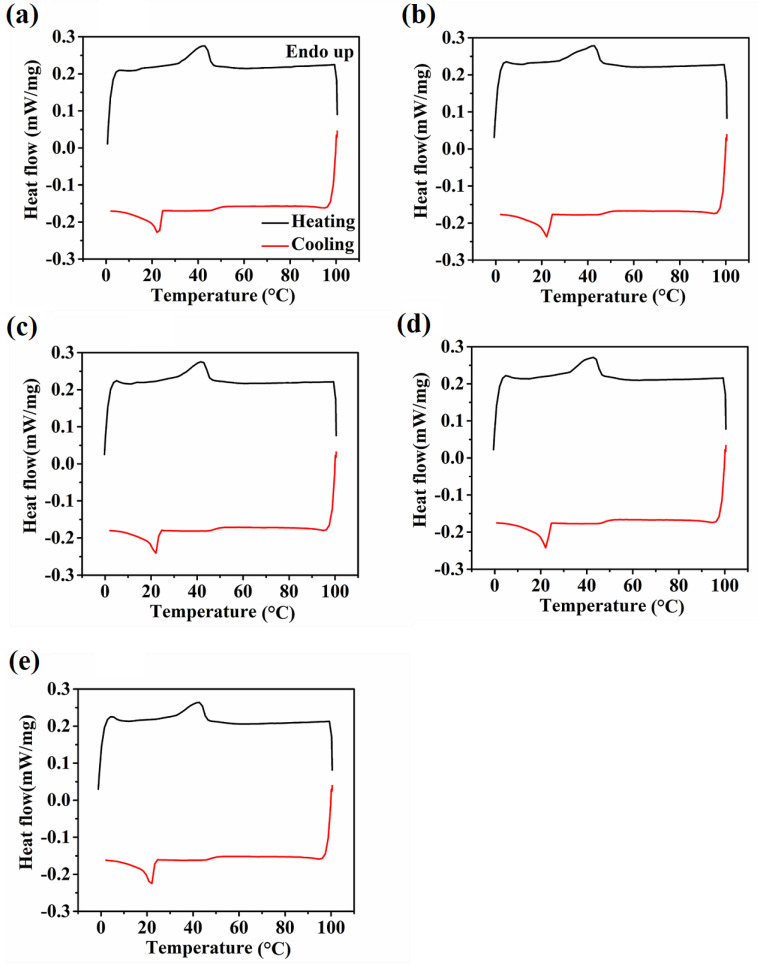
Thermograms of oleogel samples: (**a**) SE0, (**b**) SE1, (**c**) SE3, (**d**) SE5, and (**e**) SE10.

DSC studies help determine the crystallization temperature and melting point of the oleogels [66]. The thermograms of all the samples in the temperature range of 0 °C and 100 °C are represented in Figure 7. The variations in the thermal events can be linked to the physical changes in the oleogels throughout the melting (endothermic) and cooling (exothermic) processes. It has been reported that the melting temperature (T_m_) and crystallization temperature (T_c_) of SW are 43.92 and 38.49 °C, respectively [21]. In our study, one prominent signal was observed at ~43 °C in the heating cycle for each sample. There was a slight decrease in the T_m_ of SW-based oleogels compared to the neat wax. This can be attributed to the colligative properties of waxes, which help them solubilize in vegetable oils. Studies have also reported that the temperatures of wax-based oleogels are frequently lower in comparison to neat waxes [67]. The area under the crystallization and melting curve gives an idea about the enthalpy of the samples. The T_m_ of the oleogels were in the order of SE0 (42.97 °C) > SE5 (42.87 °C) > SE10 (42.70 °C) > SE1 (42.40 °C) > SE3 (41.60 °C). It was observed that there was a decrease in T_m_ from SE0 to SE3, but an increment in SE5 was shown (Table 2). Thus, it is evident that the addition of SYL in higher concentrations led to temperature variations in T_m_. Similarly, the melting enthalpy (∆H_m_) of the prepared oleogel samples followed a similar pattern. ∆H_m_ is a unit of measurement for the amount of energy required to change the SW crystals to liquid. It varied between 0.32 and 0.42 mW/mg. Apart from SE0, the rest samples (SE1, SE3, SE5, and SE10) had lower ∆H_m_ and T_m_ values, indicating the reduction in their thermal strength. 

The cooling or crystallization curve of the samples also followed a similar trend to the endothermic process. Understanding the thermal behavior of mixed oleogels can be aided by knowing when the crystallization process initiates [68]. The crystallization peaks of all the samples were nearly equal. The cooling cycle showed only one prominent peak at ~22 °C, suggesting it to be the T_c_ of the prepared oleogels. Therefore, we can hypothesize that an increase in SYL concentration does not affect the crystallization process of the oleogels. Compared to the thermal study of neat SW, there is a shift in the crystallization curve of the prepared oleogels, suggesting that the presence of RSO and SYL can affect the crystallization process of the SW crystals [21]. Studies have also reported that the presence of phospholipids can affect the microstructure and thermal properties, thereby changing the molecular assembly of the wax crystals [69]. The enthalpy of crystallization (∆H_c_) denotes the amount of energy the samples liberated to crystallize. The ∆H_c_ of SE5 had the highest value among all the samples, suggesting that it released a high amount of energy to become a stable composition. 

The degree of supercooling (∆T) is the driving force behind the nucleation and development of wax crystals [70]. Mathematically, ∆T is the difference between T_m_ and T_c_. The degree of supercooling was lower in all the SYL-containing oleogels than in the control (SE0). This advocated that nucleation induction time was delayed in the SYL-containing oleogels [71]. Among SYL-added oleogels, SE5 showed the highest ∆T, with SE3 markedly the lowest. This suggests that the nucleation process in SE3 was the slowest, which can be attributed to the slow crystallization process in SE3. The slow crystallization process promoted the formation of larger wax crystals, as observed from polarized light micrographs (Figure 3) [65]. Further, the high ∆T in SE5 resulted in the formation of smaller wax crystals than in the rest of the samples, which can be explained by the quick induction of the nucleation of the wax crystals (Table 2). The observed changes can be attributed to the interaction of the lipid molecules with lecithin which is modifying the crystallization process of the wax crystals present in the oleogel. Furthermore, the SW-based oleogels undergo a reversible melting–crystallization transition, which means that the system can revert to its former state after being subjected to higher temperatures.

As a summary of our studies, oleogel has been reported to be a possible replacement for *trans* fatty acids and saturated fatty acids-based food products. It fulfills the nutritional needs of the human body while maintaining shelf-life, texture, and sensory characteristics of the product derived using oleogel [72,73]. The study of the physicochemical properties of oleogels plays a vital role in the acceptance of oleogel-derived food products. In the present study, an in-depth analysis of RSO-SW-based oleogels with varying SYL concentrations was performed. The physiochemical behavior of prepared oleogel samples was characterized through microscopy, colorimetry, FTIR, mechanical, and DSC analysis. Bright-field and polarized light micrographs of the produced oleogels revealed the existence of needle-like wax crystals, the thickness and length of which varied with SYL concentration. The microarchitecture of SE5 showed a dense wax crystal structure. The physical appearance of the prepared oleogel was confirmed to be a light shade of yellow by the colorimetry study. To a certain extent, the addition of SYL in the oleogel did not affect the appearance. FTIR spectra confirmed the presence of several functional groups in the oleogel samples, which were also present in the raw materials. However, the hydrogen-bonding peak of SW was not observed in the oleogel samples. The mechanical study suggested that the inclusion of SYL increases the firmness and rigidity of the prepared oleogels. The stress–relaxation study parameter (i.e., F_0_ and F_60_) values revealed that the SE5 sample showed a better relaxation profile and mechanical properties than the rest of the oleogel samples. DSC thermograms of the oleogel samples revealed that the T_m_ and Tc were found to be ~43 °C and ~22 °C, respectively. The ΔH_c_ was highest in SE5. This suggested that SE5 emitted the highest energy to achieve a stable composition. ΔT was significantly lower in SE3 among SYL-added oleogels, resulting in a delay in nucleation and hence the development of superior wax crystals. In conclusion, it can be stated that the addition of SYL in lower concentrations promotes the formation of larger-sized fat crystals. However, in higher concentrations, SYL encourages the formation of a dense crystal network. 

## 4. Conclusions

The present study has shown that the addition of soy lecithin can modify the microstructural and physicochemical properties of soybean oil and soy wax-based oleogels. Such modifications of these oleogels can be helpful in developing oleogel-based food products and as a way to replace the unhealthy fatty acids (namely, *trans*- and saturated fatty acids). The obtained oleogel, containing 5 mg of soy lecithin, showed dense crystal microarchitecture with good mechanical and thermal properties. The presence of such microstructure was attributed to the slow nucleation process that resulted in the formation of better and superior crystals than other lecithin-containing formulations. Such microarchitecture further showed better mechanical strength and improved stress relaxation properties. These properties indicate the physical stability of the prepared oleogels and can potentially replace many solid lipid products such as butter or shortening agents. Thus, such oleogels can be used as raw material for cooking purposes (e.g., confectionary products) that require adding a stable and structured fat with solid-like consistency. Therefore, further studies are necessary to fully understand the role and effect of such oleogels when used in food products.

## Figures and Tables

**Table 1:** Composition of prepared samples (for 40 g).

SAMPLES	RSO (g)	SW (g)	SYL Stock (g)
SE0	35.60	4.40	0.00
SE1	34.60	4.40	1.00
SE3	32.60	4.40	3.00
SE5	30.60	4.40	5.00
SE10	25.60	4.40	10.00

**Table 2:** Thermal parameters of the oleogels.

Samples	T_m_ (°C)	ΔH_m_ (mW/mg)	T_c_ (°C)	ΔH_c_ (mW/mg)	Supercooling (ΔT)
SE0	42.97	0.43	22.11	−0.24	20.86
SE1	42.40	0.38	22.12	−0.22	20.28
SE3	41.60	0.32	22.11	−0.20	19.49
SE5	42.87	0.38	22.12	−0.26	20.75
SE10	42.70	0.33	22.12	−0.23	20.58

## Data Availability

Not applicable.
